# Ageing-induced changes in the redox status of peripheral motor nerves imply an effect on redox signalling rather than oxidative damage

**DOI:** 10.1016/j.freeradbiomed.2016.02.008

**Published:** 2016-05

**Authors:** Brian McDonagh, Siobhan M. Scullion, Aphrodite Vasilaki, Natalie Pollock, Anne McArdle, Malcolm J. Jackson

**Affiliations:** MRC-Arthritis Research UK Centre for Integrated Research into Musculoskeletal Ageing, Department of Musculoskeletal Biology, Institute of Ageing and Chronic Disease, University of Liverpool, Liverpool L69 3GA, UK

**Keywords:** CP, 3-carboxy-proxyl, CPH, 1-Hydroxy-3-carboxy-2,2,5,5-tetramethylpyrrolidine, Nav1.5, Voltage gated sodium channel isoform, NCAM, Neural cell adhesion molecule, NEM, N-ethylmaleimide, NMJ, Neuromuscular junction, NOX2, NAD(P)H oxidase 2, Prdx, Peroxiredoxin, SOD1, CuZn superoxide dismutase

## Abstract

Ageing is associated with loss of skeletal muscle fibres, atrophy of the remaining fibres and weakness. These changes in muscle are accompanied by disruption of motor neurons and neuromuscular junctions although the direct relationship between the nerve and muscle degeneration is not understood. Oxidative changes have been implicated in the mechanisms leading to age-related loss of muscle mass and in degeneration of the central nervous system, but little is known about age-related changes in oxidation in specific peripheral nerves that supply muscles that are affected by ageing. We have therefore examined the sciatic nerve of old mice at an age when loss of tibialis anterior muscle mass and function is apparent. Sciatic nerve from old mice did not show a gross increase in oxidative damage, but electron paramagnetic resonance (EPR) studies indicated an increase in the activity of superoxide and/or peroxynitrite in the nerves of old mice at rest that was further exacerbated by electrical stimulation of the nerve to activate muscle contractions. Proteomic analyses indicated that specific redox-sensitive proteins are increased in content in the nerves of old mice that may reflect an adaptation to regulate the increased superoxide/peroxynitrite and maintain redox homoeostasis. Analysis of redox active cysteines showed some increase in reversible oxidation in specific proteins in nerves of old mice, but this was not universally seen across all redox-active cysteines. Detailed analysis of the redox-active cysteine in one protein in the nerve of old mice that is key to redox signalling (Peroxiredoxin 6, Cys 47) showed a minor increase in reversible oxidation that would be compatible with a change in its redox signalling function. In conclusion, the data presented indicate that sciatic nerve from old mice does not show a gross increase in oxidative damage similar to that seen in the TA and other muscles that it innervates. Our results indicate an adaptation to increased oxidation with minor changes in the oxidation of key cysteines that may contribute to defective redox signalling in the nerve.

## Highlights

• Sarcopenia is accompanied by disruption of motor neurons and neuromuscular junctions.
• Old nerves have decreased axon numbers and diameter.
• EPR studies showed increased superoxide/peroxynitrite activities in old nerves.
• Old nerves show changes in redox sensitive proteins without major oxidative damage.
• Results indicate altered redox signalling pathways in nerves from old mice.

## Graphical abstract

**Figure:**
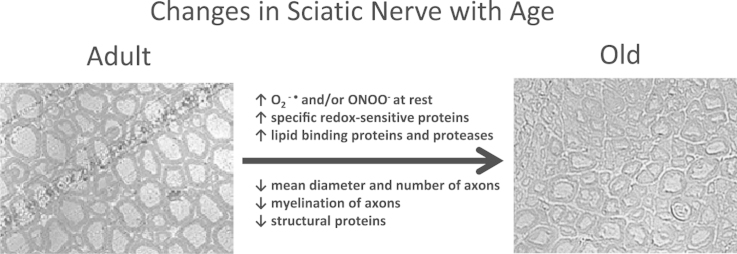

## 1 Introduction

Age-related loss of muscle fibres occurs over a substantial portion of later life and plays a major role in declining quality of life in older people [1]. Cross-sectional data from Lexell et al. [2], [3] indicate that the number of skeletal muscle fibres decreased in man from ~50 years of age and continue to decrease over subsequent years. Similar rates of loss of motor units are observed in EMG studies in ageing [4]. There is good evidence of denervation in muscle fibres from elderly subjects with fibre type grouping, accumulation of severely atrophic angular fibres, and expression of proteins associated with denervation such as neural cell adhesion molecule (NCAM) and the voltage gated sodium channel isoform (Nav1.5) by the atrophic fibres [5], [6], [7], [8], [9], [10]. There is also evidence that in both man and rodents, a reduction in the number of motor neurons occurs with ageing [10], [11]. The role of this change in innervation in age-related loss of muscle fibres is unknown, although denervation can lead to loss of muscle fibres and conversely experimental damage to muscle can lead to loss of the innervating neuromuscular junction (NMJ) (e.g. see [12], [13]).

Studies of the innervation of individual muscle fibres in aged mice have observed NMJs with a variety of age-related structural alterations, including axonal swellings, sprouting, synaptic detachment, partial or complete withdrawal of axons from some postsynaptic sites, and fragmentation of the postsynaptic specialization [14], [15]. Some recent data from rodents also indicate that despite the degeneration and loss of peripheral axons that occurs with aging, the number of motor neuron cell bodies in the lumbar spinal cord is relatively spared suggesting that changes predominantly occur in the periphery of motor units [14].

Although recent data indicate that reactive oxygen species (ROS)-induced oxidative damage is not the fundamental cause of ageing (or more precisely, the fundamental determinant of lifespan) [16], [17], tissues of all aged organisms are reported to contain increased oxidative damage to lipids, DNA and proteins [18], [19], [20]. Many studies have reported that mitochondrial ROS generation is increased during ageing [21] and this occurs in association with impaired mitochondrial function, oxidative damage and a change in mitochondrial redox potential [22], [23].

A mechanistic link between chronic oxidative stress *in vivo* and a loss of muscle mass and force is supported by studies of mice deficient in CuZn-superoxide dismutase (SOD1) (*Sod1*^−/−^ mice), a major antioxidant enzyme. Skeletal muscles from young *Sod1*^−/−^ mice display elevated oxidative damage to proteins, lipids, and DNA compared with those of age-matched wild-type (WT) mice and muscle masses are significantly lower than those of WT mice as early as 6 months of age [12]. Muscle mass in the *Sod1*^−/−^ mice is further reduced with age, and, by 20 months, hindlimb muscle mass in *Sod1*^−/−^ mice is nearly 50% lower than that in age-matched WT mice [12]. Furthermore, we have shown that restoration of CuZnSOD only in neurons of *Sod1KO* mice prevented the increased muscle mitochondrial ROS production, premature muscle atrophy, and weakness [24] suggesting that altered redox status in the motor neuron is likely an initiating event responsible for the neuromuscular declines observed early in life in *Sod1KO* mice and potentially during normal aging.

A number of studies have examined the effect of age on oxidative stress and redox regulation in the central nervous system e.g. [25], [26], but data on the redox status of the peripheral nervous system during ageing is sparse. In the light of the evidence that changes in redox homoeostasis in motor neurons may occur in the periphery of motor units, the aim of the current work was to use novel experimental approaches to examine the redox homoeostasis of peripheral nerves of wild type mice at the age when loss of muscle fibres and weakness occurs. We hypothesised that the sciatic nerve of mice would show age-related changes in the activities of specific reactive oxygen species and redox homoeostasis that may contribute to loss of neuron function and loss of muscle innervation.

## 2 Methods

### 2.1 Mice

Adult (6–8 months) and old (26–28 months) male WT C57Bl6 mice and adult mice were fed on a standard laboratory diet and subjected to a 12-h light/dark cycle. Experiments were performed in accordance with UK Home Office guidelines under the UK Animals (Scientific Procedures) Act 1986 and received ethical approval from the University of Liverpool Animal Welfare Committee. The aesthetic used varied depending up on the experimental design. Mice were anesthetized with pentobarbital sodium, with an initial dose of 65 mg/kg body mass for adult mice and 20 mg/kg for old mice via an intraperitoneal injection, supplemental doses were administered as required to maintain a depth of anaesthesia sufficient to prevent response to tactile stimuli. In other experiments old and adult mice were anesthetised using gas anaesthesia, in 2% isoflurane in 2 l/min oxygen delivered via a precision vaporizer.

### 2.2 In vivo contraction protocol

Each age cohort was split into three equal groups: (i) control mice, which did not receive the contraction protocol (unstimulated); (ii) mice that received the contraction protocol and an infusion of 1-Hydroxy-3-carboxy-2,2,5,5-tetramethylpyrrolidine (CPH) was started immediately following the end of the contractions, mice were sacrificed at 2 h post contractions; and (iii) mice that received the contraction protocol and allowed to recover for 24 h before commencing the 2 h CPH infusion. For the mice in groups ii and iii, both hindlimbs were subjected to a 15-min period of nerve stimulation to generate isometric contractions of the hindlimb muscles [27]. The stimulation protocol used was a square wave pulse of 0.2 ms duration at 60 V and a frequency of 100 Hz contracted every 4 s and repeated 180 times. Pentobarbital sodium was used as the anaesthetic throughout and mice were sacrificed by administration of an overdose of anaesthetic at the times described above and the sciatic nerves were immediately dissected.

### 2.3 Sciatic nerve dissection and analysis

The nerve was followed distally towards the tibial and common fibular nerve branches. The nerve was then followed proximally, using the scissors to break through the sciatic notch, tracing the nerve back to its five vertebral roots (L4-S3). The sciatic nerve was then dissected at the post-vertebral level and at a random point pre-branching of the tibial and common fibular branches, removing minor branches and connective tissue along the nerve.

For structural examination, sciatic nerves from 7 mice at each age were left overnight in a solution of 4% PFA and 2% glutaraldehyde. The following day the nerves were washed twice with PBS and tissues were incubated for 3 h at room temperature in 1% osmium tetroxide (OsO_4_) and 0.1 M PBS. Extensive washing with distilled water was followed by an overnight incubation in 1% uranyl acetate at room temperature. The next day tissues were dehydrated in ethanol for 5 min and subsequently washed in ethanol. Post-dehydration, tissues underwent a series of resin infiltration: 10% for 1 h, 50% resin for 2 h, 75% resin for 1 h, 100% resin for 1 h, ending by embedding in fresh 100% resin cured in a 60 °C oven for 72 h.

The resin-embedded nerve was cut using Leica EM UC6 Microtome to create a protruding square approximately 350×350 µm^2^ by use of a glass blade. Semi-thin sections were then obtained using a diamond blade, immersed in water and mounted on a microscope slide. A Ziess Axio Imager M1 was used to take randomised 100×100 µm^2^ images of sections following staining with 1% toluidine blue. Images were analysed using image analysis software (AxioVision LE). Markings of length and area were manually created, with subsequent calculation of values by the AxioVision LE software.

### 2.4 In vivo electron paramagnetic resonance analysis of the sciatic nerve

In order to define the activities of specific ROS (superoxide and/or peroxynitrite) in the sciatic nerve prior to, and following contractile activity, an *in vivo* electron paramagnetic resonance (EPR) spin probe approach was used [28]. Anesthetised mice (*n*=7 for each age and time point) were given an intravenous infusion of 1-Hydroxy-3-carboxy-2,2,5,5-tetramethylpyrrolidine (CPH; 9 mg/kg body weight bolus followed by 0.225 mg/kg/min over 2 h) via the tail vein and after 2 h the mice were killed and the sciatic nerve rapidly dissected and stored under liquid nitrogen until analysis. Samples were examined under liquid nitrogen and the stable 3-carboxy-proxyl radical formed by reaction of CPH with superoxide or peroxynitrite determined by EPR (Bruker *e-scan* benchtop spectrometer) [28]. The general settings for low temperature measurements were: microwave frequency 9.78 GHz, modulation frequency 86 kHz, modulation amplitude 6.15 G, gain 10^3^. Upon oxidation CPH is converted to the stable CP radical (3-carboxy-proxyl), standard solutions of CP were used to relatively quantify superoxide/peroxynitrite generated in the sciatic nerve [28].

### 2.5 Proteomic analyses of sciatic nerve

Sciatic nerve samples from adult (*n*=4) and old (*n*=4) mice were prepared for proteomic analysis and quantification using a label-free quantitative proteomic approach that includes a differential cysteine labelling step as recently described [29]. The approach allows simultaneous identification of up- and down regulated proteins between samples using global label free proteomics. In addition a targeted analysis of redox sensitive cysteine residues allows the identification and relative quantification of the reversible oxidation state of susceptible redox cysteine residues within samples. The proteomics analysis was performed as previously described [30]. The data-dependent label-free analysis was performed using an Ultimate 3000 RSLC™ nano system (Thermo Scientific, Hemel Hempstead, UK) coupled to a QExactive™ mass spectrometer (Thermo Scientific). The sample (5 µL corresponding to 250 ng of protein) was loaded onto the trapping column (Thermo Scientific, PepMap100, C18, 75 μm×20 mm), using partial loop injection, for 7 min at a flow rate of 4 μL/min with 0.1% (v/v) TFA. The sample was resolved on the analytical column (Easy-Spray C18 75 µm×500 mm×2 µm column) using a gradient of 97% A (0.1% formic acid) 3% B (99.9% ACN 0. 1% formic acid) to 60% A 40% B over 120 min at a flow rate of 300 nL/min. The programme used for data acquisition consisted of a 70,000 resolution full-scan MS scan (AGC set to 10^6^ ions with a maximum fill time of 250 ms) the 10 most abundant peaks were selected for MS/MS using a 17,000 resolution scan (AGC set to 5×10^4^ ions with a maximum fill time of 250 ms) with an ion selection window of 3 m/z and a normalized collision energy of 30. To avoid repeated selection of peptides for MS/MS the programme used a 30 s dynamic exclusion window.

Raw spectra were converted to mascot generated files (.mgf) using Proteome Discoverer software (Thermo Scientific). The resulting mgf files were searched against the Uniprot Mouse database sequence database (12/05/2012, 16376 sequences) using an in-house Mascot server (Matrix Science, London, UK). Search parameters used were: peptide mass tolerances, 10 ppm; fragment mass tolerance, 0.01 Da, 1+, 2+ and 3+ ions; missed cleavages, 1; instrument type, ESI-TRAP. Variable modifications included were: d(0) N-ethylmaleimide (NEM), d(5) NEM, mono-, di- and tri-oxidation of cysteine residues and oxidation of methionine and a false discovery rate of<1%. Label-free relative quantification software PEAKS™ 7 (Bioinformatics Solutions Inc, Waterloo Canada) was used to analyse RAW data files against the same mouse protein database for identifications with Mascot [31]. Proteins were considered significantly changed between adult and aged samples using a −10log *P* score>20 (equivalent to a *P* value<0.01) and using a quality value of >0.5. The full list of identified proteins including statistical analysis of protein and peptide features is included in Supplementary Files 1 and 2. Cysteine containing peptides detected with identical amino acid sequences, and both d(0) and d(5) NEM modifications independently with an individual peptide ion Mascot score of>20 were considered redox peptides. Redox peptides detected from Proteome Discovery analyses of RAW files were selected for targeted analysis using m/z data and retention times with the open software Skyline™ [32]. Targeted analysis applying m/z, retention times and fragmentation spectra for peptide selection allowed the estimation of the reduced:oxidized ratio (or d(0) / d(5) NEM) of the Cys residues using the individual parent ion intensities with Skyline™. The individual reduced:oxidized ratio for redox Cys peptides in each sample was used to calculate an average ratio of reduced:oxidized calculated for the specific cysteine residues. Intensities of targeted peptides are included in Supplementary File 3.

### 2.6 Western blotting for the protein carbonyl and peroxiredoxin 5 and 6 content of sciatic nerve

Western blotting techniques were used to assess the protein carbonyl and peroxiredoxin (Prdx) 5 and 6 contents of sciatic nerve (*n*=4) following homogenisation as previously described [24].

### 2.7 Statistical analyses for structural analysis and blotting

Data are presented as means±SEM for each experiment. Comparisons were performed by 1-way ANOVA followed by the *post hoc* LSD test. Values of *P*<0.05 were considered statistically significant.

## 3 Results

**Fig. 1:**
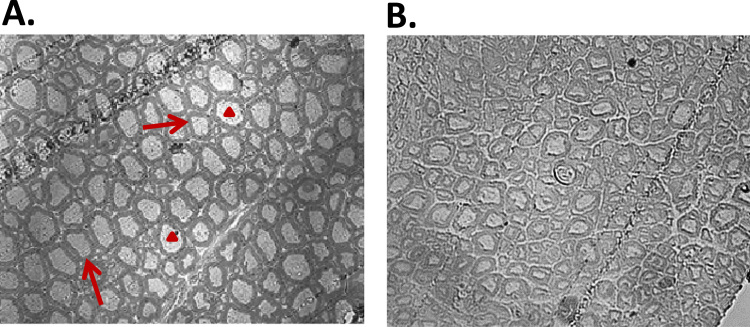
Example semi-thin sections from transverse sections of the sciatic nerves of adult (A) and old (B) mice (*n*=7). Myelin sheaths are indicated by arrows and axons by arrowheads.

In previous studies examining the effect of ageing in C57Bl6 mice, we have shown a reduced mass of the hind limb muscles, such as the tibialis anterior (TA) muscle at 26−28 months of age (e.g. see [33]) and examination of the morphological structure of the sciatic nerve that innervates these muscles showed that, compared with adult mice, those at 26−28 months had disruption of the normal axonal structure. Example semi-thin transverse sections are shown in Fig. 1. Analysis of the cross sections from mice at both ages revealed a decrease in the mean diameter of axons, a decrease in the number of axons present within each field and a reduced myelination of axons (Table 1).

**Fig. 2:**
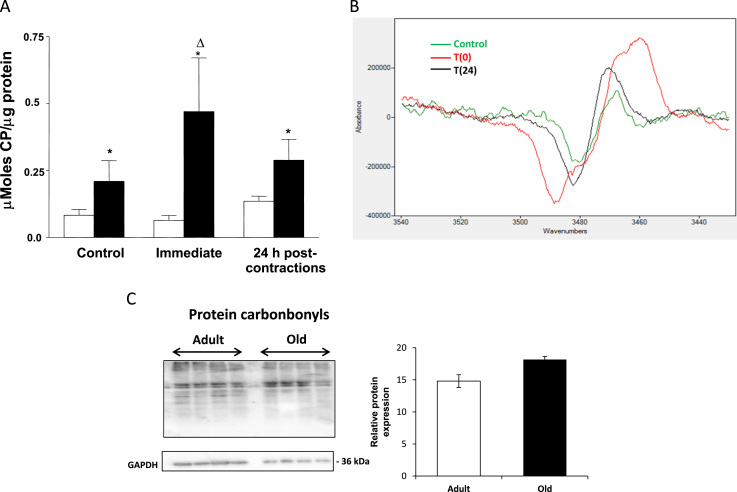
(A) Intensity of EPR signals derived from sciatic nerves of adult (open bars) and old (filled bars) mice (*n*=7). The CPH probe was infused via the tail vein over 2 h to mice at rest (Control), over 2 h following a 15 min period of isometric contractions (Immediate) or over the period 22−24 h post-contractions (24 h). * *P*<0.005 compared with values from adult mice at the same time point; ^Δ^*P*<0.05 compared with values in same group at the previous time point. (B) Representative EPR signals from sciatic nerve of old mice prior (green line) immediately after a period of isometric contractions (red line) and 24 h later (black line). (C) Western blot analysis of protein carbonyl content of sciatic nerve from old and adult mice. (For interpretation of the references to colour in this figure legend, the reader is referred to the web version of this article.) (For interpretation of the references to colour in this figure legend, the reader is referred to the web version of this article.)

In order to study the reactive species present in sciatic nerves of adult and old mice, an EPR and spin probe approach was used. The data shown in Fig. 2A and B show increased oxidation of the CPH probe infused into old mice at rest compared with adult mice. Fig. 2B shows representative images of EPR spectra from the nerves of old mice during and following contractile activity. In the 2 h period following contractile activity, adult mice showed no further increase in oxidation of the CPH probe, but a significant increase was observed post-contractions in old mice. By 24 h post-contractions oxidation in nerves of old mice had returned to pre-contraction levels. No changes were seen following contractile activity in the sciatic nerves of adult mice. The protein carbonyl content of sciatic nerves was analysed by western blotting as a marker of oxidative damage, but no significant differences were seen between the intensity of bands obtained from nerves of adult and old mice at rest (Fig. 2C).

**Fig. 3:**
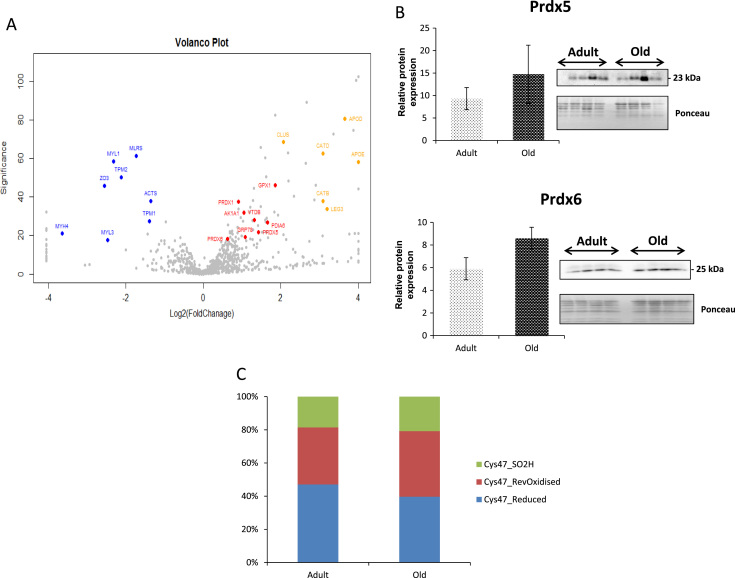
(A) Volcano plot showing changes in protein contents between sciatic nerves from old compared with adult mice detected by PEAKS label-free quantification software. Key structural proteins that are down-regulated in old mice are shown in blue and proteins involved in redox regulation that are up-regulated are shown in red. Proteins associated with neurodegenerative diseases and up-regulated with age are highlighted in orange. Proteins highlighted are ACTS (Actin alpha), AK1A1 (Alcohol dehydrogenase [NADP+], APOD (Apolipoprotein D), APOE (Apolipoprotein E), CATB (Cathepsin B), CATD (Cathepsin D), CLUS (Clusterin) GPX1 (Glutathione peroxidase 1), GRP78 (78 kDa glucose-regulated protein), LEG3 (Galectin-3), MLRS (Myosin regulated light chain 2), MYH4 (Myosin-4), MYL1 (Myosin light chain 1), MYL3 (Myosin light chain 3), PDIA6 (Protein disulphide isomerase A6), PRDX1 (Peroxiredoxin 1), PRDX5 (Peroxiredoxin 5), PRDX6 (Peroxiredoxin 6), TPM1 (Tropomyosin alpha-1 chain), TPM2 (Tropomyosin beta chain), VTDB (Vitamin D-binding protein) and ZO3 (Tight junction protein ZO-3). (B) Western blot analysis of the peroxiredoxin 5 and 6 contents of sciatic nerves from adult and old mice (± SEM). (C) The proportion of Cys47 of peroxiredoxin 6 in the reversibly oxidised, reduced and sulfinic forms from adult and old mice.

Global proteomic analysis of the nerves showed significant changes in abundance of a number of proteins. These are shown as a “volcano plot” in Fig. 3A with selected protein identified with a score −10log* P*>20 (equivalent to *P*<0.01). A total of 862 proteins were identified from adult and old sciatic nerves and the full list is included in Supplementary File 2. Proteins that were significantly decreased in content in the nerves from old mice were primarily structural proteins reflecting the extensive changes in axonal structure seen in Fig. 1 (highlighted in blue), a number of proteins showing an increased content have previously been identified as up regulated in brain during aging and in neurodegenerative diseases [34], including Apolipoproteins D and E, Cathepsin B and D and Galectin 3 (highlighted in orange). There was also an increase in the content of proteins known to function in regulation of reactive oxygen species and protein thiol homoeostasis (highlighted in red). In order to investigate the quantification from the mass spectrometry data, two proteins involved in the regulation of redox homoeostasis that had an increased content in the nerves from old mice Prdx5 (*P* value=0.0003) and Prdx6 (*P* value=0.007) were also examined by western blotting (Fig. 3B). This showed a trend towards an increased content with ageing for both proteins, but did not reach significance.

Table 2 shows the proteins identified in the sciatic nerve that contained cysteines in the reduced (NEM labelled) and reversibly oxidised (D5-NEM labelled) forms. Redox-sensitive cysteines were identified by mass spectrometry and the changes in the relative proportion of those cysteines found in the reduced or oxidised form in adult and old mice can be estimated. A number of these cysteines showed substantially greater oxidation in the samples from old compared with adult mice including Cys114 of Annexin A6, Cys254 of Creatine kinase b, Cys47 of Prdx6 and Cys49 of pyruvate kinase M1/M2. This relative oxidation was not universal and the reverse changes (i.e. greater proportion of the reduced form) were also seen in key proteins including Cys163 of lactate dehydrogenase, Cys85 of Cytochrome bc1 complex subunit 1, Cys336 of Nucleolar GTP binding protein 1 and Cys260 of the Serine protease inhibitor A3K. The majority of the redox sensitive cysteines identified are in structural or cytoskeletal regulatory proteins including Actin alpha (Cys287) and cytoplasmic (Cys257), Galectin-1 (Cys61), Gelsolin (Cys280 & 670), Myosin regulatory light chain (Cys128), Neurofilament light (Cys323) and heavy chains (Cys225, 246, 263 & 409), Periaxin (Cys86) Periperin (Cys328), Plastin (Cys336), Synaptic vesicle membrane protein (Cys99), Tubulin (Cys315/316, 376 & 303) and Vimentin (Cys328). It is also interesting to note that a number of these structural proteins also changed in abundance between sciatic nerves of adult and old mice (highlighted in Table 2).

The 1-Cys peroxiredoxin, Prdx6, was identified as containing a redox sensitive cysteine residue (Cys47). Prdx6 has both peroxidase and phospholipase activities, the peroxidase activity of this protein relies on the redox state of Cys47, which was examined in greater detail as shown in Fig. 3C, Cys47 was identified in the reduced, reversibly oxidised and the sulfinic (−SO_2_H) forms. Sciatic nerves from old mice showed a ~10% increase in the reversibly oxidised form of Cys47 compared with those from adult mice. Other regulatory proteins identified as containing redox sensitive cysteines include Rab GDP dissociation inhibitor (Cys202) and Rho GDP dissociation inhibitor (Cys79), that regulate both Rab and Rho proteins respectively, involved in membrane and intracellular vesicular trafficking and are highly expressed in the spinal cord [35], [36].

## 4 Discussion

Substantial loss of neuromuscular structure and function has been shown to occur during ageing that presents as loss and atrophy of muscle fibres, a decline in the integrity of NMJ and loss of motor units [4], [10]. Muscles that are innervated by the sciatic nerve in mice are substantially affected by aging with a loss of numbers of fibres and atrophy and weakness of the remaining fibres [19]. This is associated with disruption of some NMJ and the substantial degenerative changes in axons seen in Fig. 1, but the relationship between the nerve and muscle changes is not understood in detail nor is whether the changes in both tissues occur independently or whether degeneration of one tissue leads to the other (e.g. functional denervation leads to atrophy of muscle fibres). At the ages studied here, skeletal muscles of old mice contain increased amounts of oxidative damage including increased protein carbonyls [29], [37], but no evidence for this was found in the sciatic nerve despite the substantial disruption of nerve structure that was observed. In order to investigate whether more subtle changes in oxidation occurred in the nerve of old mice, we therefore used both EPR and redox proteomics approaches.

Infusion of the spin probe via the tail vein and subsequent analysis of the EPR signal from excised tissues has previously been used to demonstrate increased activity of reactive oxygen species in old rodents [28]. The CPH spin probe used reacts predominantly with superoxide or peroxynitrite species and is partially cell permeable. The signal obtained is therefore assumed to arise from both intracellular and extracellular oxidation [38]. CPH oxidation was found to be increased in the sciatic nerves of old mice at rest compared with nerves from adult mice indicating an increased activity of superoxide and/or peroxynitrite. Following 15 min of stimulation of muscle contractions, the CPH oxidation further increased in sciatic nerve from old mice, but no significant effect on CPH oxidation in adult mice was seen (Fig. 2A and B). The spectra obtained (Fig. 2B) are derived from superoxide and/or peroxynitrite reaction with CPH, but the current data do not permit an evaluation of the likely source of the superoxide or peroxynitrite activity detected at rest or the increase that occurred only in nerve of old mice with activation. It seems unlikely that activation of the nerve would directly stimulate intrinsic ROS generation from the tissue only in old mice and we speculate that the increase is likely to be related to increased pro-inflammatory response in old mice during extensive activity as suggested by [39]. As previously stated, the CPH probe can react with ROS in both intracellular and extracellular sources and hence the increase is compatible with an increase in circulating inflammatory mediators.

Proteomic analyses revealed a number of changes in protein content in nerves from old mice compared with those from adult mice including a substantial number of proteins that either increased or decreased in abundance (Fig. 3A). The proteins showing a significant decrease in content were predominantly structural proteins and a number of redox sensitive cysteine residues were identified in those proteins. Proteins that had a significant increased content included a number of lipid binding proteins and proteases that have been identified to be increased in mammalian brains during aging and neurodegenerative disesases including the proteases Cathepsin B and D, and also the lipid binding proteins, Apolipoprotein D and E, [40], [41], [42]. Cathepsin D knockout mice die from a neurodegenerative liposomal storage disease [43] and it has also been reported that the lipid binding protein Apo E is a substrate of the cysteine protease Cathepsin D [34], [44]. These lysosomal proteases have also been implicated in the degradation of α-Synuclein linked to the neuropathology of Parkinson’s disease [45]. The glycoprotein Galectin-3 was also up regulated, this protein induces fibrosis in animal models and has been proposed as a biomarker for prognosis and management in heart failure [46], [47]. Interestingly a number of proteins involved in regulation of cellular redox processes (i.e. Prdx5, Prdx6, Prdx1 protein disulphide isomerase and glutathione peroxidase 1, see Supplementary file 2) were also detected as up regulated by the proteomic approach. An increase in the content of such “antioxidant” enzymes is widely recognised to occur in response to increased activity of ROS in muscle tissue [19], [48] and in nerves following exposure to neurotrophic factors [49] and we speculate that these changes reflect an adaptation in old mice to increased ROS activity in the nerve that we observed with the EPR experiments (Fig. 2).

We attempted to confirm the proteomic changes in the peroxiredoxins using western blotting, but although the content in tissues from old mice showed a trend towards an increase in abundance, the increase was not statistically significant (Fig. 3). The proteomic approach used also provided information on the redox status of specific cysteine groups in proteins [50] and a list of the proteins with redox-active cysteines detected in the nerves is shown in Table 2. This table also shows the relative proportion of the specific cysteine group present in the reduced or reversibly oxidised form in nerve tissue from old compared with adult mice. Multiple proteins involved in metabolism, cytoskeleton, calcium sensitivity and antioxidant enzymes contain redox sensitive cysteine residues, whose redox state dictate activity and function and a number of the proteins identified showed an increased proportion of the cysteine in the oxidised form (e.g. Cys114 of Annexin A6, Cys254 of Creatine kinase b, Cys263 of Neurofilament heavy polypeptide, Cys47 of Prdx6 and Cys49 of Pyruvate kinase M1/M2). However, this pattern was not universally found and some cysteines showed a greater proportion in the reduced form. Structures or molecules in close proximity to the site of ROS generation are most likely to undergo redox modifications and redox modifications in key metabolic processes can alter a wide variety of downstream protein targets, influencing a variety of key regulators of distinct post translational modifications such as phosphorylation, acetylation and ubiquitination. Transient oxidation and reduction of key cysteines in regulatory proteins is a key aspect of redox signalling processes and can effect activity [50], [51] which may be especially important when dealing with proteins with more than one function and where changes in the redox state of a particular cysteine residue may result in the gain or loss of one of these activities. The variable changes in oxidation status seen in redox-active cysteines between nerves from old and adult mice is likely to reflect modified signalling rather than any indication of an overt, age-related increase in oxidation in this tissue.

Prdx6 is a key protein involved in regulation of reactive oxygen species and in redox signalling. It is a 1-cysteine peroxiredoxin in comparison to the 2-cysteine peroxiredoxins (Prdx’s 1-5), that require a structurally close resolving cysteine and the subsequent reduction of the disulfide bond generated by thioredoxin for completion of their catalytic cycles. The phosphorylation of T177 of human Prdx6 also confers a second activity on the protein, as a Ca^2+^-independent Phospholipase A2 through which it has been reported to activate plasma membrane NAD(P)H oxidase (NOX2) [52]. Prdx6 has been described as playing a role in the repair of oxidised phospholipids in cell membranes [53] and if the phospholipase activity is inhibited, it has been reported to help prevent lung damage after hypoxia [54]. The yeast orthologue of this 1-Cys peroxiredoxin, Prx1p, can be resolved and maintained in its active peroxiredoxin form by physiological concentrations of reduced glutathione indicating a robust recycling mechanism [55]. Therefore, although the current analysis of the redox status of the catalytic cysteine (Cys47) indicated no increased formation of the irreversibly oxidised sulfinic form, an increased proportion was present in the reversibly oxidised form (Fig. 3C), suggesting a sustained mild reversible oxidation. Such changes may have downstream redox signalling effects, for example, a loss in peroxidase activity but gain in phospholipase and NOX2 activities.

Thus in conclusion, the sciatic nerves from old mice are structurally small but do not show an age-related gross increase in oxidative damage in a similar manner to the muscles they innervate. However, EPR studies indicated an increase in the activity of superoxide and/or peroxynitrite occurs in the nerve of old mice at rest that was further exacerbated by electrical stimulation of the nerve to activate muscle contractions. Proteomic analyses indicated that specific redox sensitive proteins are increased in content in the nerves of old mice that may reflect an adaptation to regulate the increased superoxide/peroxynitrite and maintain redox homoeostasis. Targeted analysis of the redox active cysteines of proteins show some increase in reversible oxidation in nerves of old mice, but this was not universal across all redox-active cysteines. Subtle changes in the oxidative state of key redox regulated proteins in the nerves of old mice would be compatible with a reduced capacity to participate in redox signalling in response to stimulation.

## Figures and Tables

**Table 1:** Quantification of changes in sciatic nerve structure from old and adult mice.

	**Adult**	**Old**
**Axon diameter (µm)**	7.9±0.11	6.99±0.10*
**Axon number**	131±21	113±17*
**Myelin area (µm**^**2**^**)**	36.24±0.78	25.53±0.70*

**Table 2:** Redox sensitive targets with relative quantification of oxidative state of susceptible Cys residues and relative protein abundance, proteins in bold also change in abundance.

**Accession**	**Protein**	**Adult Vs. aged**	**(-)10log *P***	**Redox Cys**	**Adult Red/Ox**	**Aged Red/Ox**
P68254	**14-3-3 protein theta (Ywaq)**	1.00:1.59	21.52	Cys134	4.38	4.12
P63101	14-3-3 protein zeta/delta (Ywhaz)	1.00:1.34	11.31	Cys25	3.64	3.16
				Cys94	4.81	4.41
P68134	**Actin, alpha skeletal muscle (Acta1)**	1.00:0.4	59.6	Cys287	7.53	3.90
P60710	Actin, cytoplasmic 1 (Actb)	1.00:1.69	10.82	Cys257	12.96	10.24
P17183/2	Gamma-enolase (Eno1)/Alpha-enolase (Eno1)	1.00:1.41	8.84	Cys399	3.09	2.58
P07724	Albumin serum (Alb)	1.00:1.78	45.23	Cys58	0.82	1.34
				Cys77	0.02	0.03
				Cys289	0.02	0.02
				Cys500/501	0.03	0.01
P14824	Annexin A6 (Anxa6)	1.00:1.14	2.99	Cys114	9.54	8.44
Q61878	**Bone marrow proteoglycan (Prg2)**	0.00:0.100	36.64	Cys198/202	3.15	2.44
P10605	**Cathepsin B (Ctsb)**	1.00:8.49	30.49	Cys105/108	0.15	0.13
Q04447	Creatine kinase B-type (Ckb)	1.00:0.68	0.06	Cys254	1.25	1.16
Q9CZ13	Cytochrome b-c1 complex subunit 1, mitochondrial (Uqcrc1)	1.00:0.46	16.83	Cys380	1.57	1.99
Q80UW2	F-box only protein 2 (Fbxo2)	1.00:0.94	1.88	Cys75	6.42	5.96
				Cys79	0.10	0.11
P16045	Galectin-1 (Lgals1)	1.00:1.97	14.51	Cys61	4.14	3.28
Q6PAC1	Gelsolin, isoform CRA_c (Gsn)	1.00:1.38	17.23	Cys280	7.92	7.28
				Cys670	130.47	135.52
P16858	Glyceraldehyde-3-phosphate dehydrogenase (Gapdh)	1.00:0.96	1.23	Cys245	6.02	5.46
P62874	Guanine nucleotide-binding protein subunit beta-1 (Gnb1)	1.00:0.89	2.45	Cys148/149	22.75	11.48
P06151	l-lactate dehydrogenase A chain (Ldha)	1.00:1.54	11.12	Cys163	2.13	1.90
P16125	l-lactate dehydrogenase B chain (Ldhb)	1.00:1.65	9.36	Cys163	2.13	1.90
**P97457**	**Myosin regulatory light chain 2, skeletal muscle isoform (Mylpf)**	1.00:0.32	31.83	Cys128	2.93	1.79
F7A3A1	Neurofilament heavy polypeptide (Nefh)	1.00:0.81	9.84	Cys225	4.26	4.16
				Cys246	1.91	1.94
				Cys263	9.44	7.21
				Cys409	6.11	6.98
P08551	Neurofilament light polypeptide (Nefl)	1.00:0.80	10.71	Cys323	5.34	2.88
Q99ME9	Nucleolar GTP-binding protein 1 (Gtpbp4)	1.00:1.67	12.49	Cys336	13.15	15.35
E9QQ57	Periaxin (Prx)	1.00:1.13	5.08	Cys86	3.56	4.06
G5E846	Peripherin (Prph)	1.00:0.74	13.04	Cys148	2.85	2.39
				Cys328	3.45	2.77
P17742	Peptidyl-prolyl cis-trans isomerase A (Ppia)	1.00:1.11	2.26	Cys62	1.85	1.81
O08709	**Peroxiredoxin 6 (Prdx6 )**	1.00:1.54	21.46	Cys47	1.36	1.05
Q61233	**Plastin 2 (Lcp1)**	1.00:3.20	48.17	Cys336	0.22	0.42
P52480	**Pyruvate kinase isozymes M1/M2 (Pkm2)**	1.00:0.56	28.18	Cys49	4.20	3.90
P50396	Rab GDP dissociation inhibitor alpha (Gdi1 )	1.00:1.28	7.7	Cys202	4.40	3.80
Q99PT1	**Rho GDP-dissociation inhibitor 1 (Arhgdia)**	1.00:1.72	25.46	Cys79	10.32	12.91
P07759	Serine protease inhibitor A3K (Serpina3k)	1.00:1.21	2.05	Cys260	4.13	5.47
P16546	Spectrin alpha chain brain (Sptan1)	1.00:0.88	1.24	Cys956	4.20	3.90
				Cys1454	3.56	2.90
Q8VDN2	Sodium/potassium-transporting ATPase subunit alpha−1 (Atp1a1)	1.00:0.96	1.21	Cys249	3.52	3.37
Q62465	**Synaptic vesicle membrane protein VAT−1 homologue (Vat1)**	1.00:2.31	32.94	Cys99	0.84	0.98
H7BXC3	Triosephosphate isomerase (Tpi1)	1.00:1.49	8.47	Cys136	3.77	2.72
P68369/8	Tubulin alpha−1 A chain (Tuba1a)/Tubulin alpha−4 A chain (Tuba4a)	1.00:0.78	7.26	Cys315/316	3.85	2.02
				Cys376	1.16	1.05
				Cys303	5.50	5.40
Q9R0P9	Ubiquitin carboxyl-terminal hydrolase isozyme L1 (Uchl1)	1.00:1.14	2.75	Cys152	6.00	5.78
P20152	**Vimentin OS (Vim)**	1.00:1.67	35.87	Cys328	3.91	3.59

## Footnotes

Appendix A Supplementary data associated with this article can be found in the online version at 10.1016/j.freeradbiomed.2016.02.008.

## References

[1] Sayer A.A., Robinson S.M., Patel H.P., Shavlakadze T., Cooper C., Grounds M.D. (2013). New horizons in the pathogenesis, diagnosis and management of sarcopenia. Age Ageing.

[2] Lexell J., Downham D., Sjostrom M. (1986). Distribution of different fibre types in human skeletal muscles. Fibre type arrangement in m. vastus lateralis from three groups of healthy men between 15 and 83 years. J. Neurol. Sci..

[3] Lexell J., Taylor C.C., Sjostrom M. (1988). What is the cause of the ageing atrophy? Total number, size and proportion of different fiber types studied in whole vastus lateralis muscle from 15- to 83-year-old men. J. Neurol. Sci..

[4] Campbell M.J., McComas A.J., Petito F. (1973). Physiological changes in ageing muscles. J. Neurol. Neurosurg. Psychiatry.

[5] Andersen J.L. (2003). Muscle fibre type adaptation in the elderly human muscle. Scand. J. Med. Sci. Sports.

[6] Lexell J., Downham D.Y. (1991). The occurrence of fibre-type grouping in healthy human muscle: a quantitative study of cross-sections of whole vastus lateralis from men between 15 and 83 years. Acta Neuropathol..

[7] Lexell J., Taylor C.C. (1991). Variability in muscle fibre areas in whole human quadriceps muscle: effects of increasing age. J. Anat..

[8] Urbanchek M.G., Picken E.B., Kalliainen L.K., Kuzon W.M. (2001). Specific force deficit in skeletal muscles of old rats is partially explained by the existence of denervated muscle fibers. J. Gerontol. A Biol. Sci. Med. Sci..

[9] Wang Z.M., Zheng Z., Messi M.L., Delbono O. (2005). Extension and magnitude of denervation in skeletal muscle from ageing mice. J. Physiol..

[10] Rowan S.L., Rygiel K., Purves-Smith F.M., Solbak N.M., Turnbull D.M., Hepple R.T. (2012). Denervation causes fiber atrophy and myosin heavy chain co-expression in senescent skeletal muscle. PLoS One.

[11] Tomlinson B.E., Irving D. (1977). The numbers of limb motor neurons in the human lumbosacral cord throughout life. J. Neurol. Sci..

[12] Muller F.L., Song W., Jang Y.C., Liu Y., Sabia M., Richardson A., Van Remmen H. (2007). Denervation-induced skeletal muscle atrophy is associated with increased mitochondrial ROS production. Am. J. Physiol. Regul. Integr. Comp. Physiol..

[13] Li Y., Lee Y., Thompson W.J. (2011). Changes in aging mouse neuromuscular junctions are explained by degeneration and regeneration of muscle fiber segments at the synapse. J. Neurosci..

[14] Chai R.J., Vukovic J., Dunlop S., Grounds M.D., Shavlakadze T. (2011). Striking denervation of neuromuscular junctions without lumbar motoneuron loss in geriatric mouse muscle. PLoS One.

[15] Valdez G., Tapia J.C., Kang H., Clemenson G.D., Gage F.H., Lichtman J.W., Sanes J.R. (2010). Attenuation of age-related changes in mouse neuromuscular synapses by caloric restriction and exercise. Proc. Natl. Acad. Sci. USA.

[16] Perez V.I., Bokov A., Van Remmen H., Mele J., Ran Q., Ikeno Y., Richardson A. (2009). Is the oxidative stress theory of aging dead?. Biochim. Biophys. Acta.

[17] Gems D., Doonan R. (2009). Antioxidant defense and aging in *C. elegans*: is the oxidative damage theory of aging wrong?. Cell Cycle.

[18] Drew B., Phaneuf S., Dirks A., Selman C., Gredilla R., Lezza A., Barja G., Leeuwenburgh C. (2003). Effects of aging and caloric restriction on mitochondrial energy production in gastrocnemius muscle and heart. Am. J. Physiol. Regul. Integr. Comp. Physiol..

[19] Vasilaki A., Mansouri A., Van Remmen H., van der Meulen J.H., Larkin L., Richardson A.G., McArdle A., Faulkner J.A., Jackson M.J. (2006). Free radical generation by skeletal muscle of adult and old mice: effect of contractile activity. Aging Cell.

[20] Sastre J., Pallardo F.V., Vina J. (2003). The role of mitochondrial oxidative stress in aging. Free Radic. Biol. Med..

[21] Van Remmen H., Jones D.P. (2009). Current thoughts on the role of mitochondria and free radicals in the biology of aging. J. Gerontol. A Biol. Sci. Med. Sci..

[22] Jang Y.C., Van Remmen H. (2009). The mitochondrial theory of aging: insight from transgenic and knockout mouse models. Exp. Gerontol..

[23] Dimauro I., Pearson T., Caporossi D., Jackson M.J. (2012). In vitro susceptibility of thioredoxins and glutathione to redox modification and aging-related changes in skeletal muscle. Free Radic. Biol. Med..

[24] Sakellariou G.K., Davis C.S., Shi Y., Ivannikov M.V., Zhang Y., Vasilaki A., Macleod G.T., Richardson A., Van Remmen H., Jackson M.J., McArdle A., Brooks S.V. (2014). Neuron-specific expression of CuZnSOD prevents the loss of muscle mass and function that occurs in homozygous CuZnSOD-knockout mice. Faseb J..

[25] Cahill-Smith S., Li J.M. (2014). Oxidative stress, redox signalling and endothelial dysfunction in ageing-related neurodegenerative diseases: a role of NADPH oxidase 2. Br. J. Clin. Pharmacol..

[26] Vida C., Gonzalez E.M., De la Fuente M. (2014). Increase of oxidation and inflammation in nervous and immune systems with aging and anxiety. Curr. Pharm. Des..

[27] McArdle A., van der Meulen J., Close G.L., Pattwell D., Van Remmen H., Huang T.T., Richardson A.G., Epstein C.J., Faulkner J.A., Jackson M.J. (2004). Role of mitochondrial superoxide dismutase in contraction-induced generation of reactive oxygen species in skeletal muscle extracellular space. Am. J. Physiol. Cell Physiol..

[28] Kozlov A.V., Szalay L., Umar F., Kropik K., Staniek K., Niedermuller H., Bahrami S., Nohl H. (2005). Skeletal muscles, heart, and lung are the main sources of oxygen radicals in old rats. Biochim. Biophys. Acta.

[29] McDonagh B., Sakellariou G.K., Smith N.T., Brownridge P., Jackson M.J. (2014). Differential cysteine labeling and global label-free proteomics reveals an altered metabolic state in skeletal muscle aging. J. Proteome Res..

[30] McDonagh B., Sakellariou G.K., Smith N.T., Brownridge P., Jackson M.J. (2015). Redox proteomic analysis of the gastrocnemius muscle from adult and old mice. Data Brief.

[31] Zhang J., Xin L., Shan B., Chen W., Xie M., Yuen D., Zhang W., Zhang Z., Lajoie G.A., Ma B. (2012). PEAKS DB: de novo sequencing assisted database search for sensitive and accurate peptide identification. Mol. Cell. Proteom.: MCP.

[32] MacLean B., Tomazela D.M., Shulman N., Chambers M., Finney G.L., Frewen B., Kern R., Tabb D.L., Liebler D.C., MacCoss M.J. (2010). Skyline: an open source document editor for creating and analyzing targeted proteomics experiments.

[33] Palomero J., Vasilaki A., Pye D., McArdle A., Jackson M.J. (2013). Aging increases the oxidation of dichlorohydrofluorescein in single isolated skeletal muscle fibers at rest, but not during contractions. Am. J. Physiol. Regul. Integr. Comp. Physiol..

[34] Elliott D.A., Tsoi K., Holinkova S., Chan S.L., Kim W.S., Halliday G.M., Rye K.A., Garner B. (2011). Isoform-specific proteolysis of apolipoprotein-E in the brain. Neurobiol. Aging.

[35] Boureux A., Vignal E., Faure S., Fort P. (2007). Evolution of the Rho family of ras-like GTPases in eukaryotes. Mol. Biol. Evol..

[36] Stenmark H. (2009). Rab GTPases as coordinators of vesicle traffic. Nat. Rev. Mol. Cell. Biol..

[37] Vasilaki A., Simpson D., McArdle F., McLean L., Beynon R.J., Van Remmen H., Richardson A.G., McArdle A., Faulkner J.A., Jackson M.J. (2007). Formation of 3-nitrotyrosines in carbonic anhydrase III is a sensitive marker of oxidative stress in skeletal muscle. Proteom. Clin. Appl..

[38] Kozlov A.V., Szalay L., Umar F., Fink B., Kropik K., Nohl H., Redl H., Bahrami S. (2003). Epr analysis reveals three tissues responding to endotoxin by increased formation of reactive oxygen and nitrogen species. Free Radic. Biol. Med..

[39] Della Gatta P.A., Cameron-Smith D., Peake J.M. (2014). Acute resistance exercise increases the expression of chemotactic factors within skeletal muscle. Eur. J. Appl. Physiol..

[40] Chan E.S., Chen C., Cole G.M., Wong B.S. (2015). Differential interaction of Apolipoprotein-E isoforms with insulin receptors modulates brain insulin signaling in mutant human amyloid precursor protein transgenic mice. Sci. Rep..

[41] Dassati S., Waldner A., Schweigreiter R. (2014). Apolipoprotein D takes center stage in the stress response of the aging and degenerative brain. Neurobiol. Aging.

[42] Fonovic M., Turk B. (2014). Cysteine cathepsins and their potential in clinical therapy and biomarker discovery. Proteom. Clin. Appl..

[43] Ketscher A., Ketterer S., Dollwet-Mack S., Reif U., Reinheckel T. (2016). Neuroectoderm-specific deletion of cathepsin D in mice models human inherited neuronal ceroid lipofuscinosis type 10. Biochimie.

[44] Zhou W., Scott S.A., Shelton S.B., Crutcher K.A. (2006). Cathepsin D-mediated proteolysis of apolipoprotein E: possible role in Alzheimer’s disease. Neuroscience.

[45] McGlinchey R.P., Lee J.C. (2015). Cysteine cathepsins are essential in lysosomal degradation of alpha-synuclein. Proc. Natl. Acad. Sci. USA.

[46] de Boer R.A., van Veldhuisen D.J., Gansevoort R.T., Muller Kobold A.C., van Gilst W.H., Hillege H.L., Bakker S.J., van der Harst P. (2012). The fibrosis marker galectin-3 and outcome in the general population. J. Intern. Med..

[47] de Boer R.A., Lok D.J., Jaarsma T., van der Meer P., Voors A.A., Hillege H.L., van Veldhuisen D.J. (2011). Predictive value of plasma galectin-3 levels in heart failure with reduced and preserved ejection fraction. Ann. Med..

[48] McArdle A., Pattwell D., Vasilaki A., Griffiths R.D., Jackson M.J. (2001). Contractile activity-induced oxidative stress: cellular origin and adaptive responses. Am. J. Physiol. Cell. Physiol..

[49] Gabaizadeh R., Staecker H., Liu W., Van De Water T.R. (1997). BDNF protection of auditory neurons from cisplatin involves changes in intracellular levels of both reactive oxygen species and glutathione. Brain Res. Mol. Brain Res..

[50] McDonagh B., Sakellariou G.K., Jackson M.J. (2014). Application of redox proteomics to skeletal muscle aging and exercise. Biochem. Soc. Trans..

[51] Marino S.M., Gladyshev V.N. (2010). Cysteine function governs its conservation and degeneration and restricts its utilization on protein surfaces. J. Mol. Biol..

[52] Krishnaiah S.Y., Dodia C., Feinstein S.I., Fisher A.B. (2013). p67(phox) terminates the phospholipase A(2)-derived signal for activation of NADPH oxidase (NOX2). Faseb J..

[53] Li H., Benipal B., Zhou S., Dodia C., Chatterjee S., Tao J.Q., Sorokina E.M., Raabe T., Feinstein S.I., Fisher A.B. (2015). Critical role of peroxiredoxin 6 in the repair of peroxidized cell membranes following oxidative stress. Free Radic. Biol. Med..

[54] Benipal B., Feinstein S.I., Chatterjee S., Dodia C., Fisher A.B. (2015). Inhibition of the phospholipase A2 activity of peroxiredoxin 6 prevents lung damage with exposure to hyperoxia. Redox Biol..

[55] Pedrajas J.R., McDonagh B., Hernandez-Torres F., Miranda-Vizuete A., Gonzalez-Ojeda R., Martinez-Galisteo E., Padilla C.A., Barcena J.A. (2016). Glutathione is the resolving thiol for thioredoxin peroxidase activity of 1-Cys peroxiredoxin without being consumed during the catalytic cycle. Antioxid. Redox Signal..

