# Supplementary material for: Ageing-induced changes in the redox status of peripheral motor nerves imply an effect on redox signalling rather than oxidative damage
Source: Free Radic Biol Med. 2016 May;94:27–35. doi: 10.1016/j.freeradbiomed.2016.02.008 (PMC4851218; doi:10.1016/j.freeradbiomed.2016.02.008)

**Supplementary File 1:**

**Ageing-induced changes in the redox status of peripheral motor nerves imply an effect on redox signalling rather than oxidative damage.**

Brian McDonagh, Siobhan M Scullion, Aphrodite Vasilaki, Natalie Pollock, Anne McArdle and Malcolm J. Jackson

MRC-Arthritis Research UK Centre for Integrated Research into Musculoskeletal Aging (CIMA), Skeletal Muscle Pathophysiology Research Group, Institute of Aging and Chronic Disease, University of Liverpool, Liverpool L69 3GA, UK.

MS Data Analysis and Search Parameters

| \| **Table 1.** Result filtration parameters. \| \| \| --- \| --- \| \| Retention time \| ≥0 \| \| Retention time \| ≤110 \| \| Feature fold change \| ≥1 \| \| Quality \| ≥1 \| \| Avg. Area \| ≥0E0 \| \| Charge \| ≥1 \| \| Charge \| ≤10 \| \| Confident sample number \| ≥1 \| \| With peptide ID \| true \| \| Normalization \| Use TIC \| \| Protein significance \| ≥20 \| \| Protein fold change \| ≥2 \| \| Confident unique supports \| ≥1 \| |  |
| --- | --- | --- | --- | --- | --- | --- | --- | --- | --- | --- | --- | --- | --- | --- | --- | --- | --- | --- | --- | --- | --- | --- | --- | --- | --- | --- | --- | --- | --- |
| \| **Table 2**. Statistics of Filtered Results \|  \| \| --- \| --- \| \|  \|  \|  \| \|  \|  \|  \| \|  \|  \|  \| \|  \|  \|  \| \|  \|  \|  \| |  |
| \| **Table 3.** Search Parameters \| \| --- \| \| Quantification type: Label free quantification Mass Error Tolerance: 10.0 ppm Retention Time Shift Tolerance: 1.0 min Dependent on PID: 38,42,47,39,43,50,40,61,62,63,41,49,53,44,51, 55,45,48,57,54,59,58,56 FDR Threshold: 1% Samples: 8 samples in 2 groups : Adult SN_1 Adult_SN_2 Adult_SN_3 Adult_SN_4  : Aged_SN_1 Aged_SN_2 Aged_SN_3 Aged_SN_4  Reference Sample: Adult_SN_4 (auto detected) Training Samples: Adult_SN_2, Adult_SN_3 (auto detected) \| |  |

Supp. Figure 1: Volcano plot of proteins


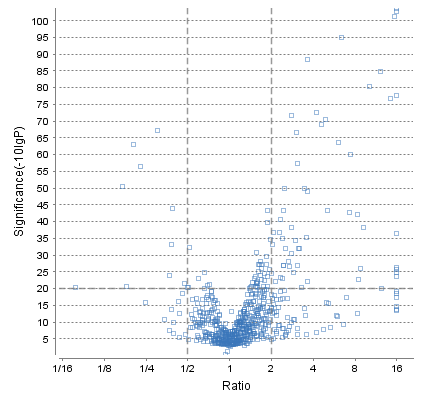


Supp. Figure 2: Volcano plot of peptides


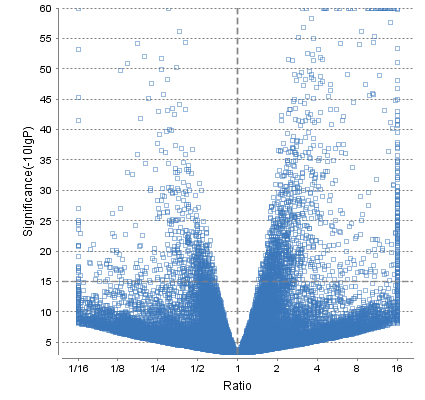


Supp. Figure 3: Distribution of vector ratio by quality


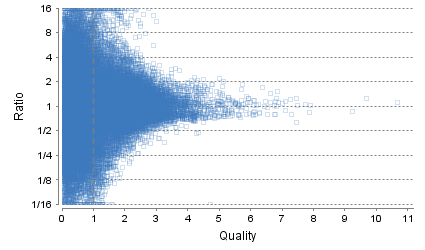


Supp. Figure 4: Distribution of vector ratio by intensity


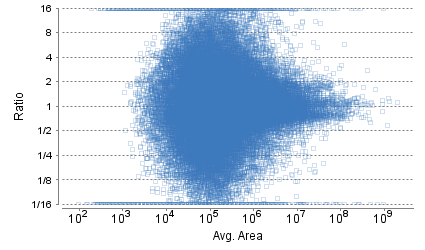


Supp. Figure 5: Retention time shift distribution


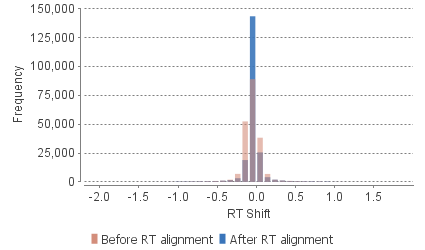


Supp. Figure 6: m/z shift distribution


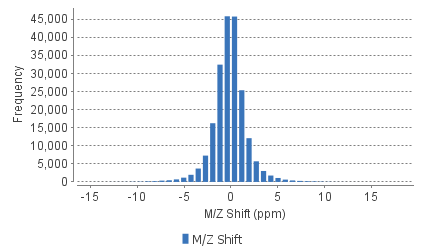

Supplement: Supplementary file 1 [file mmc1.docx]
